# Supplementary material for: Deep-Learning-Based Algorithm for the Removal of Electromagnetic Interference Noise in Photoacoustic Endoscopic Image Processing
Source: Sensors (Basel). 2022 May 23;22(10):3961. doi: 10.3390/s22103961 (PMC9147354; doi:10.3390/s22103961)
Supplement: Supplementary file 1 [file sensors-22-03961-s001.zip › sensors-1715670-supplementary.pdf]

sensors

MDPI

Article

Deep-Learning-Based Algorithm for the Removal of Electromagnetic Interference Noise in Photoacoustic Endoscopic Image Processing: Supplementary Materials

Table S1. Summary of deep-learning-based PAT studies in the literature. Here, the classification of sub-technical category in PAT was made, based on the criterion described in Ref. 9 merely for the better identification of a dedicated application in the related study.

Study	Sub-technical category in PAT	Applied dataset	Addressed issue	Applied or derived CNN architecture
[30] Song et al., 2020	PAM (OR-PAM)	In vivo mouse ear images	Accurate classification of signals between background and circulating melanoma tumor cells	VGG16-based CNN
[31] Zhang et al., 2019	PACT	PA breast images generated artificially from X-ray mammographic images	Classification and segmentation of breast cancer	GoogleNet and AlexNet
[32] Shan et al., 2019	PACT	Shepp–Logan standard test image	Correction of reflection artifacts	U-Net
[33] Tong et al., 2020	PACT	Brain, abdomen, liver cancer, and vessel images from in vivo experiments and numerical simulations	Achieving high quality image reconstruction from limited-view data with sparse measurements	Feature Projection Network (FPnet) and U-Net
[34] DiSpirito et al., 2021	PAM (OR-PAM)	In vivo mouse brain images	Achieving high resolution under undersampled condition	Fully Dense U-Net
[35] Guan et al., 2020	PACT	Circle, Shepp–Logan, vasculature, and mouse brain numerical phantom images	Removal of artifacts caused by sparse data	Fully Dense U-Net
[36] Godefroy et al., 2021	PACT	Numerical and experimental lead phantom images	Compensating for visibility artefacts caused by the limited view and bandwidth of a linear array	U-Net
[37] Chen et al., 2019	PAM (OR-PAM)	Numerical and in vivo rat brain images	Correcting motion artifacts	Customized 2-layer CNN
[38] Lan et al., 2020	PACT	Numerical phantom, chicken breast-based tissue phantom, and in vivo human palm images	Eliminating artefacts caused by the limited view and bandwidth of a linear array	Y-Net
[39] Davoudi et al., 2019	PACT	Numerical phantom and in vivo mouse whole-body images	Attaining high image quality in PACT using sparse data	U-Net
[40] Ly et al., 2022	PAM	In vivo human palm and foot images	Image segmentation	U-Net
[41] Chlis et al., 2020	PACT	In vivo human foot multispectral vasculature images	Image segmentation	Sparse-UNET (S-UNET)
[42] Lafci et al., 2021	PACT	In vivo mouse whole body images	Image segmentation	U-Net
[43] Hariri et al., 2020	PACT	Optical phantoms and in vivo mouse back images	Contrast improvement	Multi-level wavelet-CNN (MWCNN)
[44] Awasthi et al., 2020	PACT	Numerical phantoms and in vivo rat brain images	Resolution and bandwidth enhancement	U-Net
Our study	PAE	In vivo rat colorectum and rabbit urethra images	EMI noise removal	U-Net

U-Net

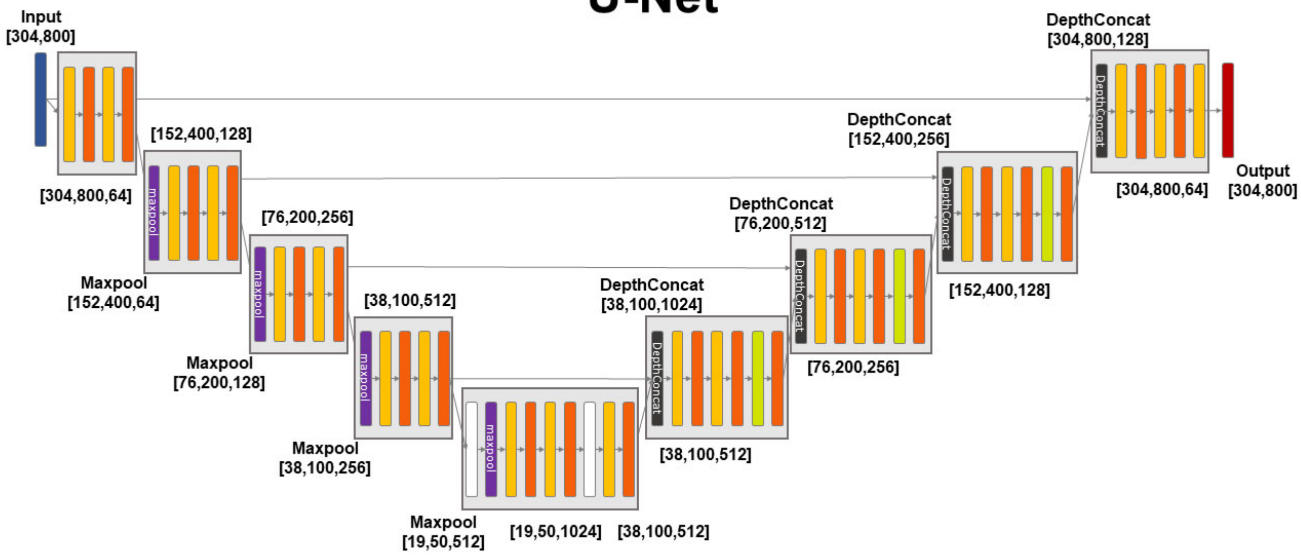
Input
[304,800]
[304,800,64]
[152,400,128]
Maxpool
[152,400,64]
[76,200,256]
Maxpool
[76,200,128]
[38,100,512]
Maxpool
[38,100,256]
Maxpool
[19,50,512]
[19,50,1024]
[38,100,512]
DepthConcat
[38,100,1024]
[38,100,512]
DepthConcat
[76,200,512]
[76,200,256]
DepthConcat
[152,400,256]
[152,400,128]
DepthConcat
[304,800,128]
[304,800,64]
Output
[304,800]
maxpool
DepthConcat

Segnet

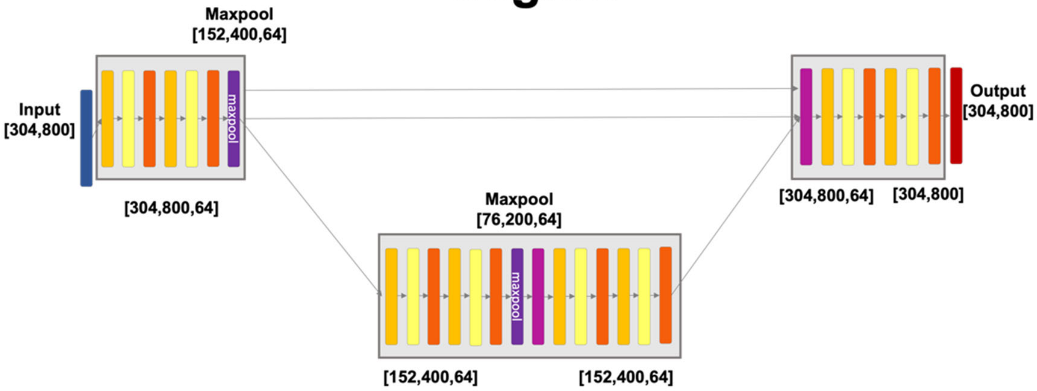
Maxpool
[152,400,64]
Input
[304,800]
[304,800,64]
Maxpool
[76,200,64]
[152,400,64]
[152,400,64]
Output
[304,800]
[304,800,64]
[304,800]
maxpool

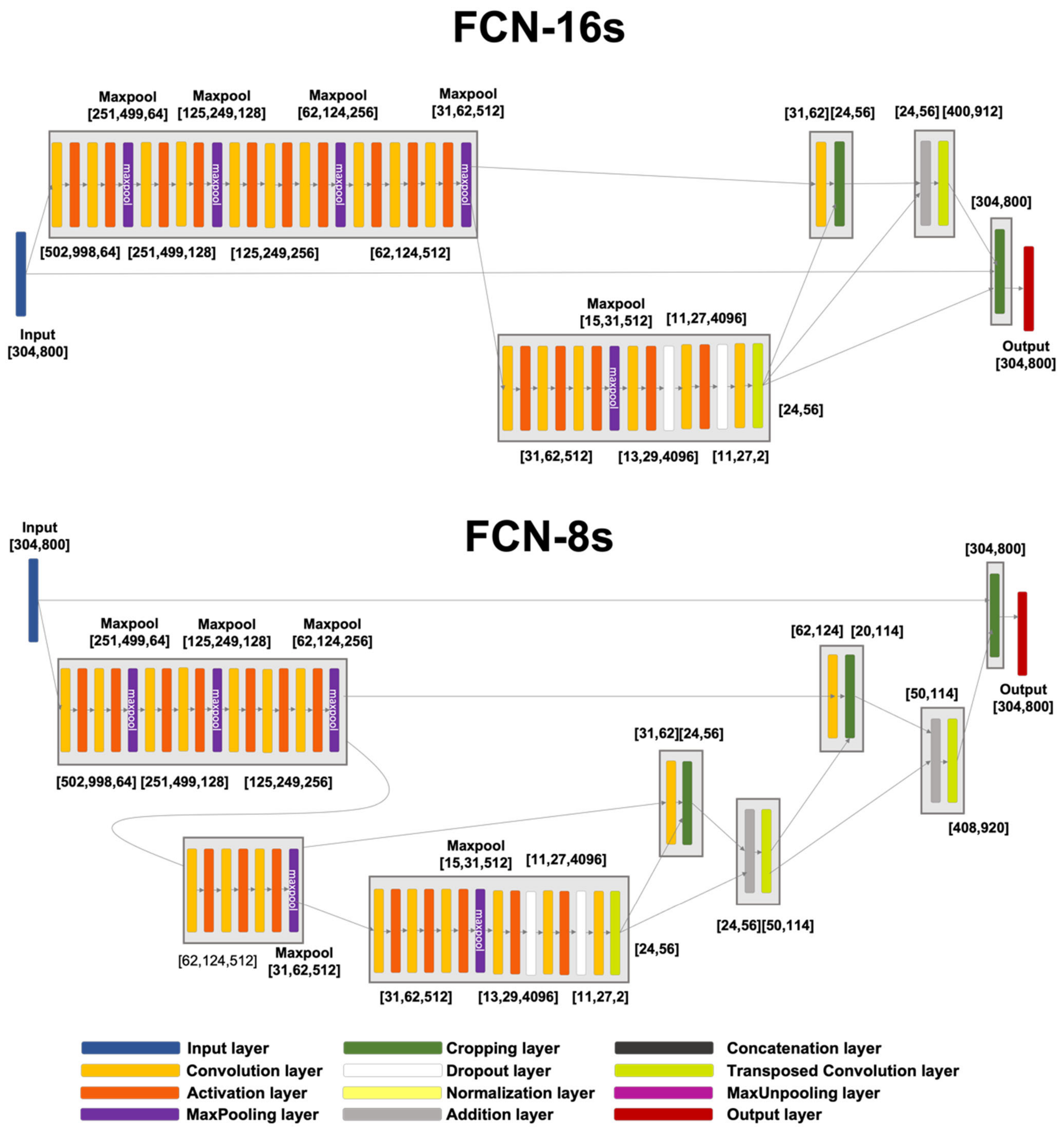

Figure S1. Neural network architecture details: U-Net, Segnet, FCN-16s, FCN-8s. Each color is assigned to a particular type of layer in the networks.

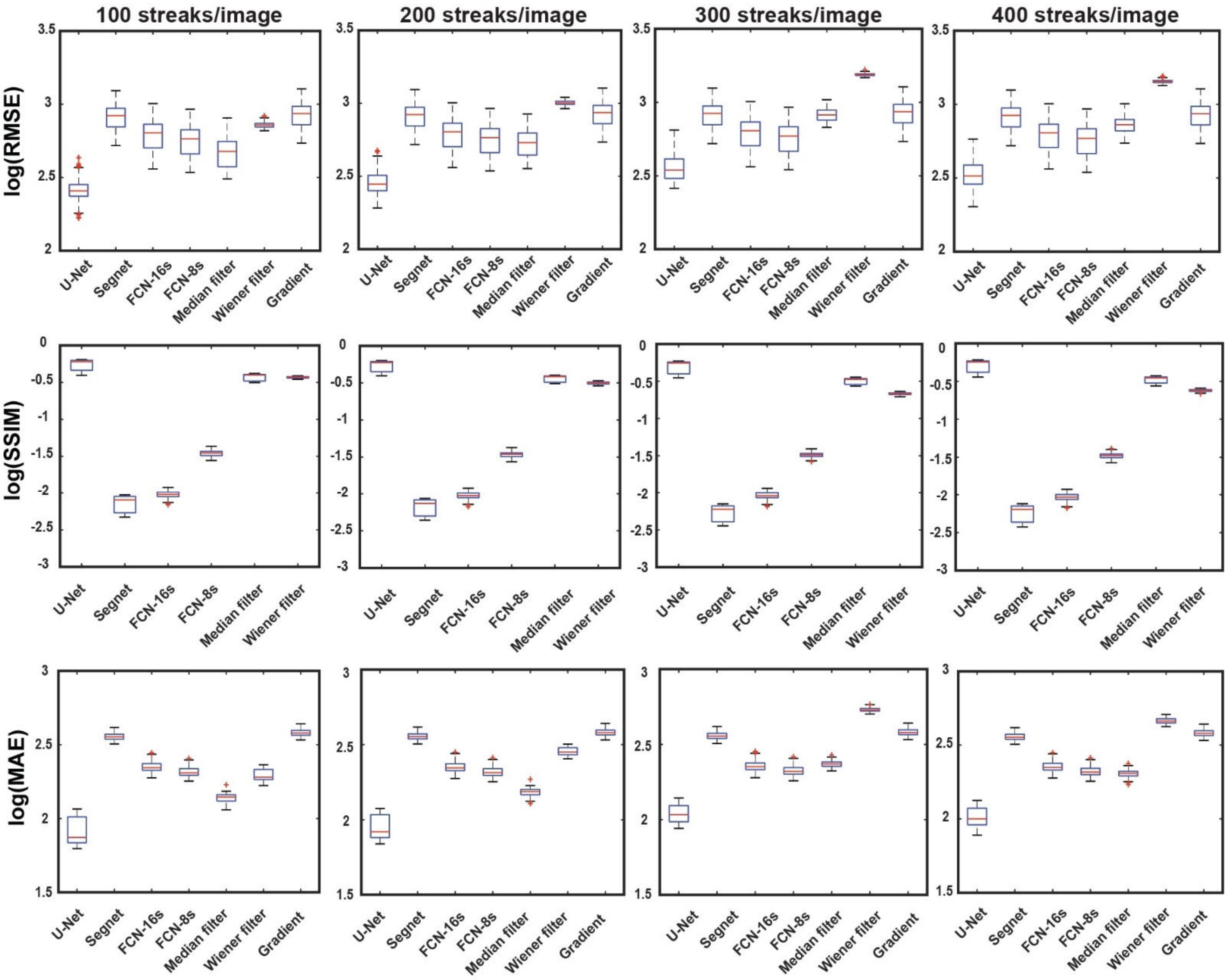

Figure S2. RMSE, SSIM, and MAE values of various denoising methods. This is to compare the four CNN-based algorithms developed in the current study and the three classical denoising methods, the median filter, Wiener filter, and the transverse signal gradient-based method utilized in [29], which were chose for comparison. The U-Net clearly presents the best noise removal results. The SSIM values for the signal gradient-based method are not shown because they were approximately 10^{-16}, almost 0, which is the worst possible value.

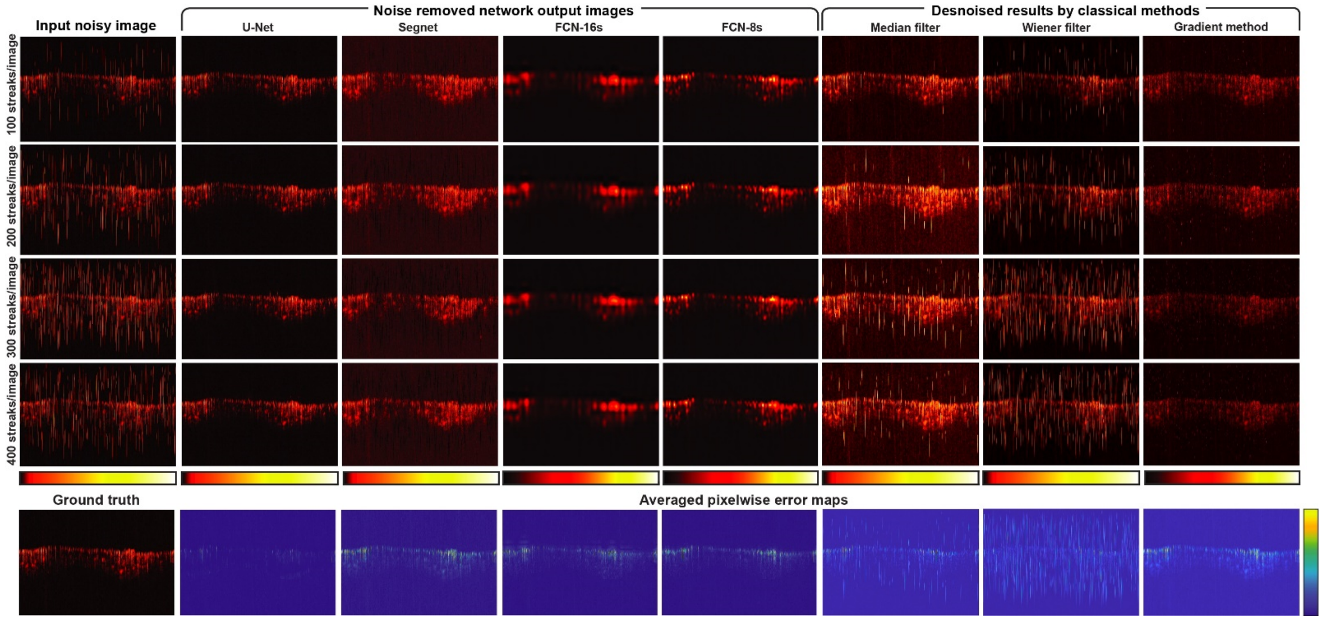

Figure S3. Comparison of the noise removal effect between the four CNN-based algorithms developed in the current study and classical denoising methods. This is an extension of Figure 4 shown in the main text, including three additional denoising results. Classical denoising algorithms, the median filter, the Wiener filter, and the transverse signal gradient-based method are presented for comparison. Here, the gradient-based method refers to the method that we developed in Ref. 29. Although this method could remove EMI noise-affected pixels very cleanly, as presented in the figure, with an involved threshold value that was set high, its main weakness was that it also removed normal capillary signals because there was quite a large overlap between the capillary signals and EMI noise in terms of pixel values and morphological features. Being aware of the issue, we initiated the current deep-learning-based study.
